# Mesencephalic Astrocyte-Derived Neurotrophic Factor Is Upregulated with Therapeutic Fasting in Humans and Diet Fat Withdrawal in Obese Mice

**DOI:** 10.1038/s41598-019-50841-6

**Published:** 2019-10-04

**Authors:** Emilia Galli, Jari Rossi, Thomas Neumann, Jaan-Olle Andressoo, Stefan Drinda, Päivi Lindholm

**Affiliations:** 1 Institute of Biotechnology, Helsinki Institute of Life Science, University of Helsinki, Helsinki, Finland; 2 Department of Anatomy, Faculty of Medicine, University of Helsinki, Helsinki, Finland; 3 Department of Internal Medicine III, Friedrich Schiller University Jena, Jena, Germany; 4 Department of Rheumatology, Cantonal Hospital St. Gallen, St. Gallen, Switzerland; 5 Department of Pharmacology, Faculty of Medicine, Helsinki Institute of Life Science, University of Helsinki, Helsinki, Finland; 6 Division of Neurogeriatrics, Department of Neurobiology, Care Sciences and Society, Karolinska Institutet, Stockholm, Sweden; 7 Hospital Buchinger-Wilhelmi, Überlingen, Germany; 8 Department for Rheumatology, Clinic St. Katharinental, Diessenhofen, Switzerland

**Keywords:** Blood proteins, Neurotrophic factors, Obesity

## Abstract

Dietary restriction induces beneficial metabolic changes and prevents age-related deterioration. Mesencephalic astrocyte-derived neurotrophic factor (MANF) shows protective effects on cells in various models of degenerative diseases. Here we studied whether circulating concentrations of MANF are associated with fasting-induced positive effects. We quantified the levels of circulating MANF from 40 human subjects before and after therapeutic fasting. As measured by an enzyme-linked immunosorbent assay (ELISA), the mean concentration of plasma MANF increased after an average fasting of 15 days. Plasma MANF levels correlated inversely with adiponectin, a hormone that regulates metabolism, thus suggesting that MANF levels are related to metabolic homeostasis. To study the effects of dietary intervention on MANF concentrations in mice, we developed an ELISA for mouse MANF and verified its specificity using MANF knock-out (KO) tissue. A switch from high-fat to normal diet increased MANF levels and downregulated the expression of unfolded protein response (UPR) genes in the liver, indicating decreased endoplasmic reticulum (ER) stress. Liver MANF and serum adiponectin concentrations correlated inversely in mice. Our findings demonstrate that MANF expression and secretion increases with dietary intervention. The MANF correlation to adiponectin and its possible involvement in metabolic regulation and overall health warrants further studies.

## Introduction

Fasting is the voluntary restriction of food intake for a defined period and can be therapeutically used for different indications^1^. Medically supervised dietary restriction (i.e., nutritional intake of 200–500 kcal/day for a period of few weeks) has been successfully used for improving conditions such as metabolic syndrome^2^. Short- to mid-term fasting therapy has been reported to decrease body mass index (BMI), glucose and insulin levels, and insulin resistance in overweight and obese subjects^3^. Fasting regulates secretion of adiponectin and leptin, which are hormone-like peptides derived from adipose tissue and maintain metabolic homeostasis^4^. Increased levels of circulating adiponectin have been reported in dietary restriction studies^5^. In general, fasting regimes induce adaptive programs to reduce metabolism in response to food deprivation, promote health, and extend lifespan in multiple model organisms^6,7^.

MANF (also known as ARMET) was originally identified based on its survival-promoting activity on cultured brain dopamine neurons^8^. Since then, the protective properties of MANF on various cell types have been demonstrated in animal models of Parkinson’s disease, cerebral ischemia, spinocerebellar ataxia, myocardial infarction, and retinal degeneration^9–14^. *Manf* conventional knock-out mice develop insulin-dependent diabetes due to postnatal loss of pancreatic insulin-producing beta cell mass, indicating that MANF is important for the function and maintenance of pancreatic beta cells^15^. Circulating MANF levels are increased in human children with recent onset of type 1 diabetes and in adults with prediabetes and type 2 diabetes^16,17^, suggesting that alterations in levels of circulating MANF can indicate changes in energy metabolism.

Subcellular MANF localizes to the ER and ER stress has been shown to induce MANF expression and secretion *in vitro* and *in vivo*^18–26^. Proliferation and survival of pancreatic beta cells in *Manf*^−/−^ mice were evidently compromised due to chronic ER stress, suggesting that MANF is important for the maintenance of ER homeostasis in the cells^15^. ER stress is caused by an imbalance between protein synthesis and protein folding capacity. To restore ER homeostasis, an adaptive unfolded protein response (UPR) is induced by the activation of inositol-requiring enzyme (IRE)1, PKR-like ER kinase (PERK), and activating transcription factor (ATF)6 pathways^27,28^. Obesity and nutrient excess are known to induce chronic ER stress in the liver and several other tissues^29^.

In a recently published study, systemic MANF was shown to have anti-aging properties related to the regulation of metabolism and immune response^30^. Using heterochronic parabiosis, which connects the blood circulation from young and old mice, the authors demonstrated that the presence of MANF in the young blood was required for the rejuvenating effect of parabiosis on age-related liver degeneration in old mice. Similarly, systemic delivery of recombinant MANF protein was able to alleviate age-related liver damage in mice^30^.

Here, we aimed to determine whether dietary restriction, a known health-promoting and anti-aging regime, affects circulating concentrations of MANF in humans. We measured plasma MANF concentrations before and after therapeutic fasting using in-lab human MANF ELISA^16^ and studied the correlations between MANF levels and biochemical and physiological parameters. To perform dietary intervention in mice, diet-induced obese (DIO) mice were subjected to dietary fat withdrawal by changing from a high-fat diet (HFD) to a normal diet (ND). We decided to use DIO mice to model the present human study population, since 75% of the human subjects in our study were overweight or obese. Changes in circulating and tissue levels of MANF and expression of UPR markers were studied in relation to diet change and subsequent weight loss in the mice. Since human MANF ELISA does not recognize mouse MANF^16^, a novel ELISA for mouse MANF protein was produced, and the specificity of the ELISA was confirmed using serum and tissue samples from MANF KO mice^15^.

## Results

### Plasma MANF is increased in fasting humans

**Figure 1:**
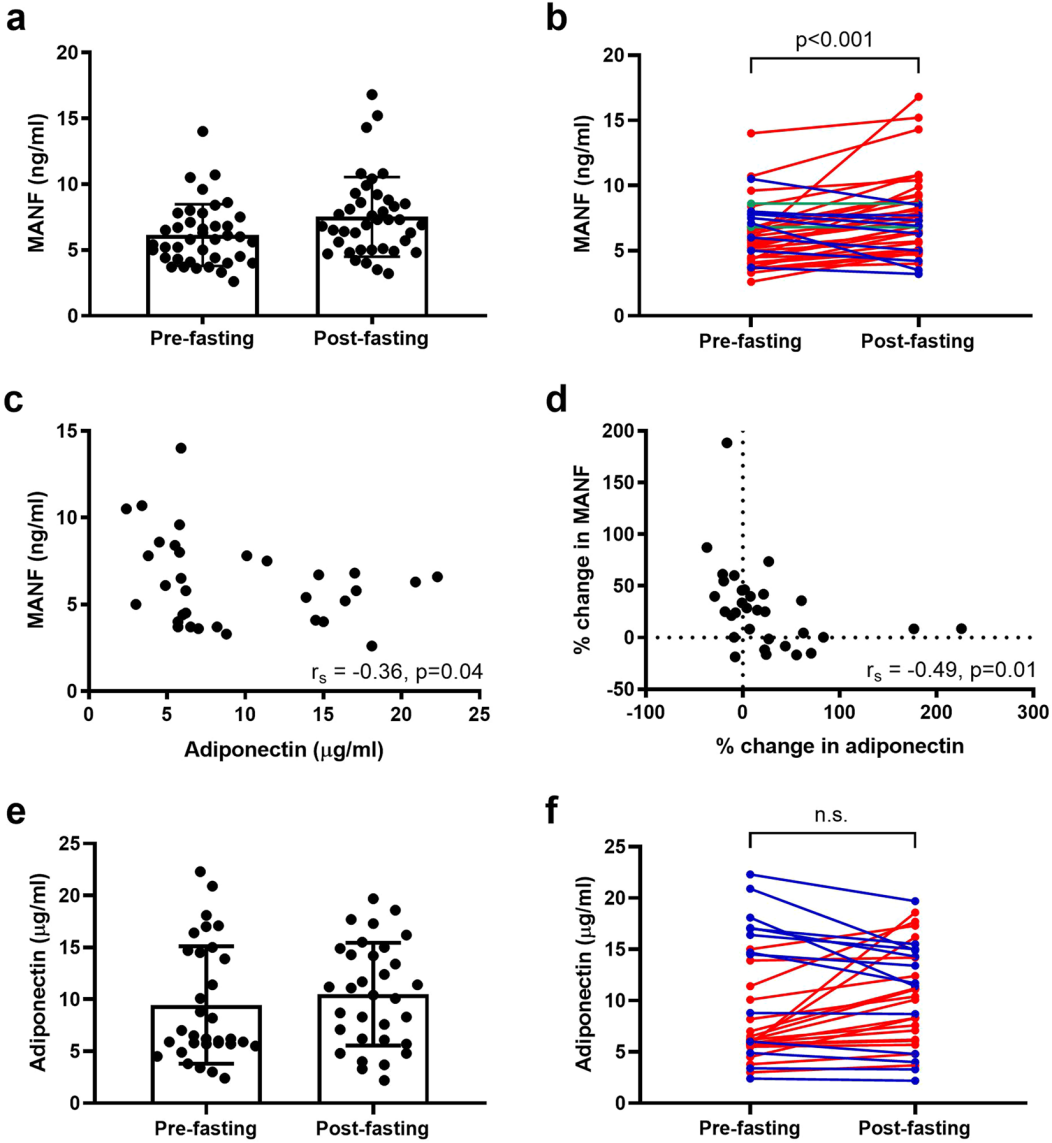
Plasma MANF levels increase with therapeutic fasting in humans. (**a**) MANF concentrations in human plasma samples by ELISA. (**b**) MANF concentration increased in 72.5% (*n* = 29/40) of cases after fasting (red lines). In 22.5% (*n* = 9/40) of cases, MANF decreased (blue lines) and in two cases (5%) remained unchanged (green lines). (**c**) Circulating concentrations of MANF (ng/ml) and adiponectin (μg/ml) correlated inversely before fasting (r_s_ = −0.36, *p* = 0.04, *n* = 32). (**d**) Percentage change in plasma MANF and adiponectin levels between pre- and post-fasting samples correlated inversely (r_s_ = −0.49, *p* = 0.01, *n* = 32). (**e**) Plasma adiponectin concentrations before and after fasting by ELISA. (**f**) Adiponectin concentration increased in 59.4% (*n* = 19/32) and decreased in 40.6% (*n* = 13/32) of cases after fasting (red and blue lines, respectively). Statistical significance was analysed by Wilcoxon signed ranks test in (b,f). Correlations were analysed by Spearman’s rank correlation in **(c**,**d**). n.s. = not significant.

The study population consisted of 40 adults with an average age of 54.5 ± 12.5 years (range 22–77 years) who volunteered to fast for therapeutic effects on various health conditions (Table 1). The average concentration of MANF in human plasma samples before fasting was 6.1 ± 2.3 ng/ml (median: 5.8 ng/ml; range: 2.6–14.0 ng/ml, Fig. 1a). In samples collected after fasting, the average MANF concentration was 7.5 ± 3.0 ng/ml (median: 7.1 ng/ml; range: 3.2–16.8 ng/ml). The mean MANF plasma concentration increased by 1.4 ng/ml (+23%) during fasting (*p* < 0.001) (Fig. 1a,b). The MANF concentration in plasma increased in 72.5% (*n* = 29/40) of the study subjects with an average increase of 2.3 ± 2.0 ng/ml (range: 0.2–11.0 ng/ml, +44%) from pre-fasting MANF concentration (Fig. 1b). In cases of decreased plasma MANF levels (*n* = 9/40, 22.5%), the average decrease was −1.2 ± 1.0 ng/ml (range: −0.1 to −3.6 ng/ml, −17%). MANF concentration was unchanged in two samples (5%).

**Table 1:** Selected background information of the human study group.

**General**	
Age, years (mean ± SD, range)	54.5 ± 12.5, 22–77
Female, *n* (%)	30 (75%)
Days of fasting (mean ± SD, range)	15.3 ± 6.3, 9–49
BMI (kg/m^2^), (mean ± SD, range)	29.8 ± 8.6, 19–61
**Diagnoses**	
Overweight, BMI 25–29.9 kg/m^2^, *n* (%)	16 (40%)
Obese, BMI ≥ 30 kg/m^2^, *n* (%)	14 (35%)
Arthritis (other than rheumatoid arthritis)	8 (20%)
Hypertonia	4 (10%)
Burn out/depression	4 (10%)
Hypothyreosis/struma	3 (7.5%)
History of cancer	3 (7.5%)

Only the most commonly occurred diagnoses are listed. BMI, body mass index.

The average duration of fasting was 15.3 ± 6.3 days (range 9–49 days, Table 1). We found no correlation between time of fasting and MANF concentration (ng/ml) after fasting (r_s_ = −0.16, p = 0.34, n = 40), or the percentage change in MANF during of fasting (r_s_ = 0.07, *p* = 0.66, *n* = 40; data not shown).

### Correlation analysis of circulating MANF with BMI, glucose, and insulin

MANF serum levels did not differ between lean (BMI < 25 kg/m^2^), overweight (BMI 25–29.9 kg/m^2^), or obese (BMI ≥ 30 kg/m^2^) subjects in pre- or post-fasting situations. The percentage change in MANF during fasting also did not differ between the different BMI groups (*p* > 0.05; data not shown). Circulating MANF concentrations showed no correlation with weight, BMI, waist circumference, or with blood lipids (Table 2). Furthermore, no correlations were observed between MANF and blood glucose or insulin levels, or with the levels measured after oral glucose tolerance test (OGTT). No correlation was found between MANF levels and homeostatic model assessment (HOMA) indexes for the estimation of insulin resistance (IR) and beta cell function (%β) (HOMA2 IR or HOMA2%β, respectively).

**Table 2:** Correlation analysis between circulating MANF levels and clinical variables.

Variable	Pre-fasting			Post-fasting			% change		
	*n*	Spearman’s Rho	*p*-value	*n*	Spearman’s Rho	*p*-value	*n*	Spearman’s Rho	*p*-value
Age	40	−0.11	0.52	40	−0.13	0.43		n.a.	n.a.
Weight	40	0.04	0.80	40	−0.08	0.65	40	−0.20	0.22
BMI	40	0.02	0.93	40	−0.03	0.87	40	−0.21	0.19
Waist circumference	39	0.09	0.59	29	0.09	0.65	29	−0.12	0.54
Fasting glucose	40	−0.27	0.09	40	−0.05	0.75	40	0.25	0.13
Glucose 0.5 h after OGTT	40	−0.16	0.32	40	0.01	0.94	40	0.02	0.89
Glucose 2 h after OGTT	40	−0.05	0.75	39	0.14	0.39	39	0.07	0.67
Fasting insulin	40	−0.03	0.88	40	0.01	0.97	40	0.14	0.39
Insulin 0.5 h after OGTT	39	0.07	0.66	40	−0.16	0.32	39	−0.17	0.31
First-phase insulin secretion	39	0.07	0.66	40	−0.10	0.56	39	−0.04	0.82
Early insulin response	39	0.10	0.56	40	−0.03	0.85	39	0.03	0.88
Oral disposition index	39	0.09	0.60	40	0.01	0.96	39	0.004	0.98
Matsudata index	39	0.04	0.82	40	0.09	0.60	39	−0.09	0.59
QUICKI	40	−0.05	0.78	40	0.19	0.24	40	−0.19	0.24
HOMA2 IR	40	−0.03	0.86	40	0.04	0.80	39	0.23	0.16
HOMA2%β	40	0.18	0.27	40	0.12	0.45	39	−0.08	0.63
Cholesterol	40	−0.20	0.22	39	−0.11	0.51	39	−0.10	0.55
LDL	40	−0.20	0.23	39	−0.12	0.48	39	−0.18	0.28
HDL	40	−0.15	0.36	39	−0.06	0.73	39	−0.07	0.66
Triglycerides	40	0.04	0.83	39	0.02	0.89	39	0.08	0.61
CRP	40	0.27	0.10	39	−0.03	0.86	38	0.08	0.61
Leukocytes	40	0.22	0.17	40	−0.04	0.79	39	−0.22	0.17
Leptin	33	0.03	0.88	33	−0.11	0.54	33	0.21	0.25
Adiponectin	32	−0.36	0.04*	33	−0.18	0.33	32	−0.49	0.01*
IGF-1	33	0.06	0.75	33	0.04	0.84	33	0.10	0.57

BMI, body mass index; CRP, C-reactive protein; HDL, high-density lipoprotein; HOMA2 IR, Homeostatic model assessment index 2 insulin resistance; HOMA2%β, Homeostatic model assessment index 2 beta cell function; IGF-1, insulin-like growth factor 1; LDL, low-density lipoprotein; OGTT, oral glucose tolerance test; QUICKI, quantitative insulin-sensitivity check index; n.a., not applicable. % change; correlation of the percentage change in MANF concentration during fasting with the percentage change in other variables.

### Pre-fasting MANF and adiponectin show an inverse correlation

We found an inverse correlation between MANF and adiponectin concentrations measured in pre-fasting plasma samples (r_s_ = −0.36, *p* = 0.04, *n* = 32, Fig. 1c). Furthermore, the percentage change in MANF concentrations during fasting correlated inversely with the percentage change in adiponectin levels (r_s_ = −0.49, *p* = 0.01, *n* = 32, Fig. 1d). In contrast to plasma MANF, average adiponectin levels measured before and after fasting (9.4 ± 5.7 μg/ml and 10.5 ± 4.9 μg/ml, respectively) did not significantly differ. Plasma adiponectin concentration was increased in 59.4% (*n* = 19/32) and decreased in 40.6% (*n* = 13/32) of the study subjects (*p* = 0.15; Fig. 1e,f). No correlations were found between the plasma levels of MANF and leptin or insulin like-growth factor-1 (IGF-1) (Table 2).

### Generation and validation of mouse MANF ELISA

To quantify endogenous MANF protein levels in mouse serum and tissues, we developed a mouse (m)MANF ELISA and tested its specificity by MANF KO mouse samples^15^. The sensitivity of the mouse MANF ELISA was 29 pg/ml. The assay detected both mouse and human MANF but did not detect homologous human cerebral dopamine neurotrophic factor (CDNF) with 59% amino acid identity^31^ and did not respond to MANF KO mouse serum or tissue lysates in contrast to samples from wild-type mice (Supplementary Table S1). The mMANF ELISA yielded a slightly different concentration-response curve for recombinant mouse (rm) and human (rh) MANF proteins (Supplementary Table S1). The endogenous MANF concentration in mouse serum samples was calculated by the standard curve of rmMANF protein whereas endogenous mouse MANF in tissue lysates was calculated by the standard curve of rhMANF. This was because mouse tissue samples only gave acceptable dilutional linearity when compared to the rhMANF standard curve (Supplementary Table S2).

Recombinant mMANF could be measured with acceptable accuracy and precision (total error within 15%) at concentrations of 31.25 to 1000 pg/ml. The average intra- and interassay variations were 5.1% coefficient of variation (CV) (range: 3.7–7.5%) and 9.7% CV (range: 5.4–16.5%), respectively (Supplementary Table S3). The dynamic range of the mMANF ELISA for rhMANF was 62.5 to 1,000 pg/ml and the average intra- and interassay variations were 9.8% CV (range: 7.5–13.0%) and 8.6% CV (range: 4.7–13.4%), respectively (Supplementary Table S3). The linearity of dilution was within 80% to 106% in mouse serum samples (*n* = 7) and 90% to 112% in mouse tissue lysates (*n* = 5) (Supplementary Table S4). The average recovery of 50 to 250 pg/ml spikes of recombinant mMANF and hMANF to study samples was 96 ± 8% in mouse sera (*n* = 18) and 99 ± 5% in tissue lysates, respectively (*n* = 6, Supplementary Table S5).

We found that mouse MANF serum concentrations measured using mouse MANF ELISA correlated positively with the absorbance values at 414 nm (Abs_414nm_), signifying the extent of haemolysis in the serum samples (r_s_ = 0.86, *p* < 0.001, *n* = 90, Supplementary Fig. S1a). By excluding mouse serum samples with Abs_414nm _≥ 0.3, the positive correlation between MANF concentrations and Abs_414nm_ was abolished (r_s_ = 0.23, *p* = 0.16, *n* = 39, Supplementary Fig. S1b), indicating that low levels of haemolysis do not dictate serum MANF concentration values. Therefore, only the MANF values that were quantified from mouse cardiac serum samples with a cut-off limit of 0.3 for Abs_414nm_ we included in the analysis.

### MANF protein levels are increased in the liver after change from high-fat to normal diet and weight loss in mice

To further investigate the changes of MANF levels in relation to diet change in a mammalian model system, we analysed MANF concentrations in serum samples from mice on diets with different fat content. A group of DIO mice was fed a HFD until 13 weeks-of-age and then switched to a ND for 2 weeks (HFD/ND), while a parallel group remained on the HFD. A control group of mice was fed a ND throughout the experiment (n = 12/group).

**Figure 2:**
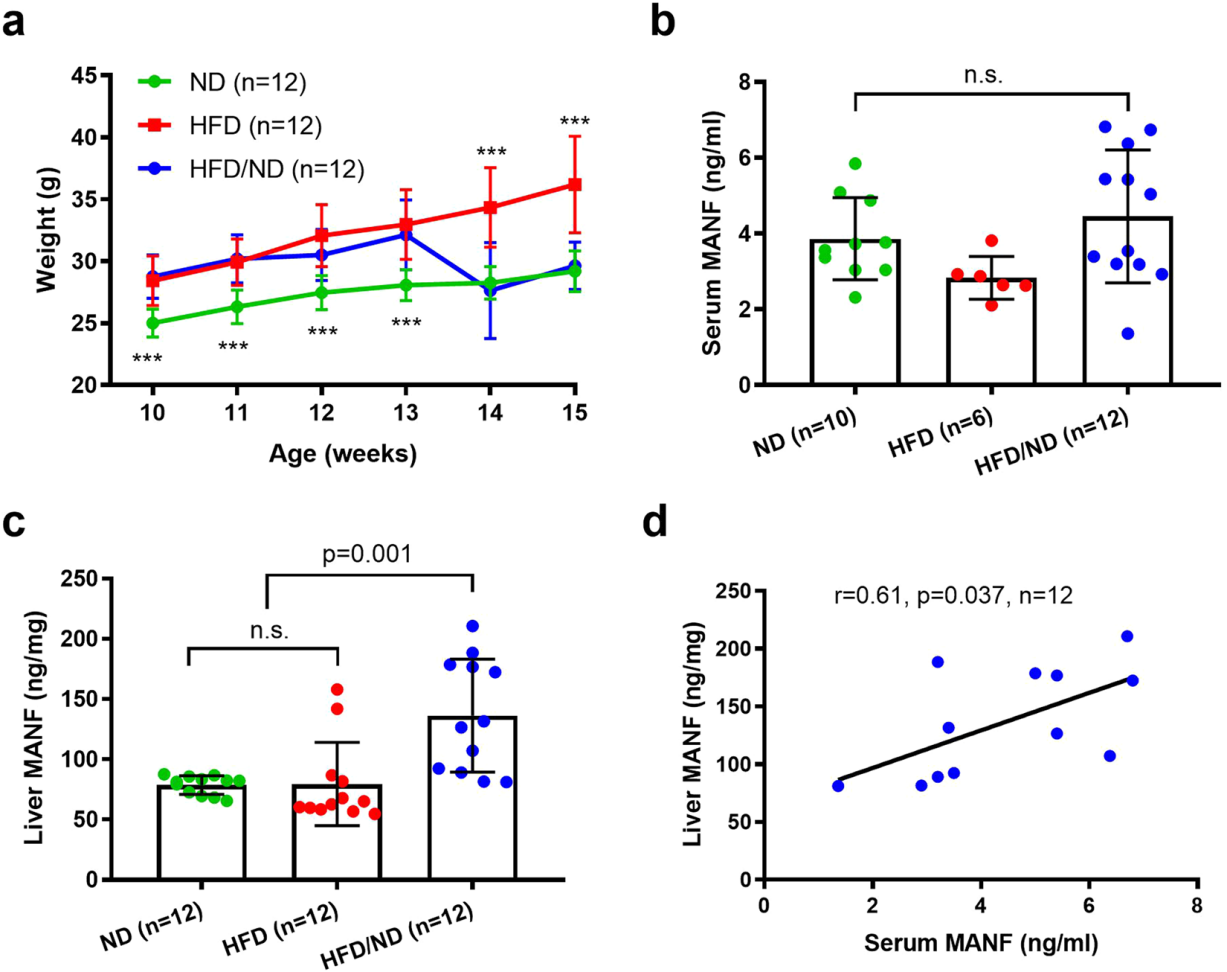
MANF concentration increases in mouse liver after change from high-fat to normal diet. (**a**) Average body weight was higher in mice on high fat diet (HFD and HDF/ND groups) than controls on a normal diet (*p* < 0.001, weeks 10–13). After change from a HFD to a ND, the HFD/ND group lost weight when compared with the HFD group (*p* < 0.001, weeks 14 and 15). The average weight of the HFD/ND group decreased similarly as the ND group (*p* = 1.0, weeks 14 and 15). (**b**) Serum MANF levels did not differ between the study groups as measured by ELISA. (**c**) MANF concentration increased in the liver of the HFD/ND group compared to the HFD or ND groups (*p* = 0.001 for both). (**d**) Serum MANF (ng/ml) and liver MANF (ng/mg total protein) correlated positively (r = 0.61, *p* = 0.037, *n* = 12) in the HFD/ND group. Statistical significance was analysed by repeated measures ANOVA followed by pairwise comparisons with Bonferroni correction in (**a**). Differences between group means were analysed by one-way ANOVA followed by Tukey HSD test in (**b**,**c**). Correlation was analysed by Pearson’s correlation in (**d**). n.s. = not significant; HFD, high-fat diet; ND, normal diet.

The average weight of the ND, HFD, and HFD/ND groups differed over time as analysed using repeated measures ANOVA (F(2,33) = 19.7, *p* < 0.001). Follow-up pairwise comparisons of the interaction with Bonferroni correction revealed that the mean weight in the HFD and HFD/ND groups fed a HFD was higher than in the ND group at the ages of 10 to 13 weeks (*p* < 0.001, Fig. 2a). After the change from HFD to ND, the mean weight of HFD/ND group was significantly lower than that of the HFD group (*p* < 0.001) and similar to that of the ND group (*p* = 1.0) as measured at the age of 14 and 15 weeks (Fig. 2a).

The MANF serum concentration in the ND group at 15 weeks-of-age was 3.86 ± 1.09 ng/ml (range: 2.32–5.85 ng/ml, *n* = 10). In the HFD group, the MANF concentration was 2.84 ± 0.57 ng/ml (range: 2.11–3.82 ng/ml, *n* = 6), while in the HDF/ND group the corresponding concentration was 4.44 ± 1.76 ng/ml (range: 1.36–6.82 ng/ml, *n* = 12, Fig. 2b). The average concentration of serum MANF was 1.62 ng/ml (i.e., 58%) higher in the HFD/ND group than the HFD group. The differences in MANF concentrations between the ND, HFD, and HFD/ND groups did not reach statistical significance (*p* = 0.076). Serum MANF concentration in the HFD/ND group did not correlate with weight (r = 0.24, *p* = 0.46, *n* = 12, data not shown) as measured at 15 weeks-of-age.

To study the effects of diet change on MANF levels in tissues which are important for systemic energy homeostasis^4^, we quantified MANF concentrations in the liver, skeletal muscle, and pancreas of the mice. MANF levels did not differ between the ND, HFD, or HFD/ND study groups in the pancreas or skeletal muscle (data not shown). However, the mean MANF concentration increased by 72% in the livers of mice from the HFD/ND group (*p* = 0.001, Fig. 2c). The liver MANF concentration in the ND and HFD group was 78.7 ± 7.7 and 79.5 ± 34.5 ng/mg total protein, respectively, while in the HFD/ND group the corresponding concentration was 136.4 ± 46.8 ng/mg total protein (*p* = 0.001, *n* = 12 in all, Fig. 2c). We observed that liver and serum MANF levels correlated positively in the HFD/ND group (r = 0.61, *p* = 0.037, *n* = 12, Fig. 2d) but not in the ND (r = 0.28, *p* = 0.43, *n* = 10, data not shown) or HFD group (r = 0.29, *p* = 0.57, *n* = 6, data not shown).

### Inverse correlation between liver MANF and serum adiponectin in mice

**Figure 3:**
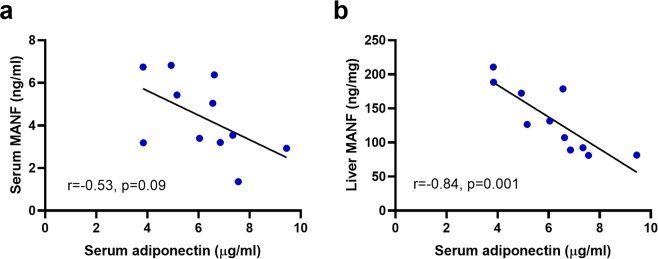
Concentrations of circulating adiponectin and liver MANF correlate inversely in mice after diet change. (**a**) Trend of inverse correlation between serum MANF and adiponectin concentrations (r = −0.53, *p* = 0.09, *n* = 11) in the HFD/ND group. (**b**) Inverse correlation between liver MANF and circulating adiponectin levels (r = −0.84, *p* = 0.001, *n* = 11) in the HFD/ND group. Correlation was analysed by Pearson’s correlation in (**a**,**b**). HFD, high-fat diet; ND, normal diet.

Serum MANF and adiponectin levels did not correlate statistically significantly in the mice of the HFD/ND group (r = −0.53, *p* = 0.09, *n* = 11, Fig. 3a). However, we found a strong inverse correlation between circulating adiponectin and liver MANF protein concentrations (r = −0.84, *p* = 0.001, *n* = 11, Fig. 3b).

### Liver MANF concentrations are associated with changes in UPR markers

We next analysed the transcript levels of UPR-related genes in liver samples from the ND, HFD, and HFD/ND groups to determine if the observed increase in liver MANF levels of the HFD/ND group was related to ongoing ER stress. In the liver samples of the obese HFD group, transcript levels of *Atf6a*, spliced X-box-binding protein 1 (*sXbp1*), activating transcription factor 4 (*Atf4*), DNA damage-inducible transcript 3 (*Ddit3*, also known as *Chop)*, and DnaJ homolog subfamily B member 9 (*Dnajb9*, also known as *Erdj4*) were increased compared to the ND group, suggesting that the three UPR sensors ATF6α, IRE1α, and PERK were activated. Activated ATF6α translocates to the Golgi where it is cleaved to produce a transcription factor that induces expression of UPR target genes, including chaperones and a pro-apoptotic transcription factor *Chop*^32,33^. *sXbp1*, generated downstream of IRE1α, induces expression chaperones and components of ER associated degradation (e.g. *Erdj4*^19^). ATF4, downstream of PERK, regulates induction of *Chop*^34^.

**Figure 4:**
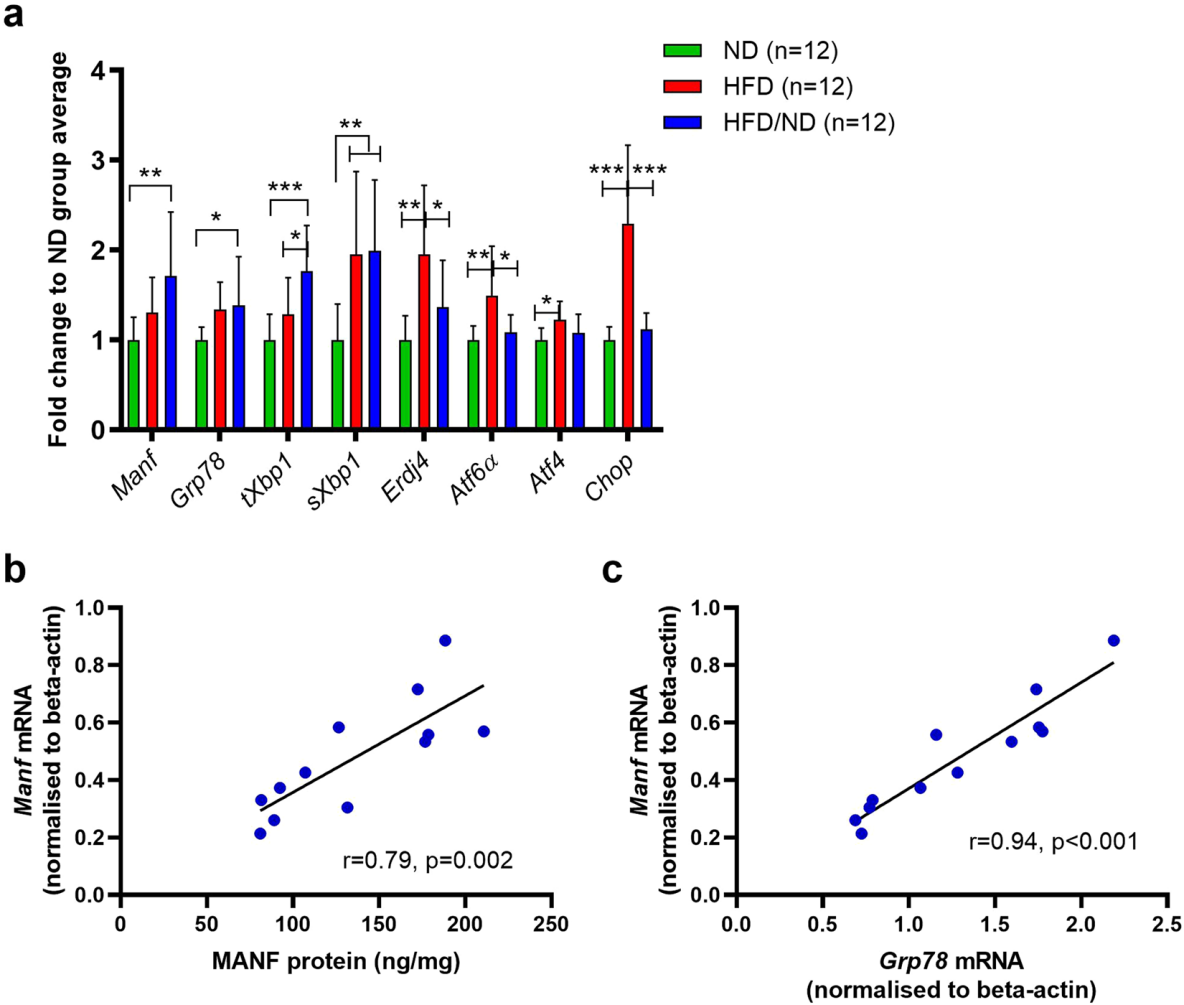
Expression of *Manf* and UPR genes in livers of mice after diet change as measured by qRT-PCR. (**a**) Expression of *Erdj4*, *Atf6α*, and *Chop* was downregulated by diet fat removal and weight loss in the HFD/ND group compared to the HFD group. (**b**) MANF protein and mRNA levels correlated positively in the HFD/ND group (r = 0.79, *p* = 0.02, *n* = 12). (**c**) *Manf* and *Grp78* mRNA levels correlated positively (r = 0.94, *p* < 0.001, *n* = 12) in the HFD/ND group. Statistical significance was analysed by one-way ANOVA followed by Tukey HSD test in (**a**). Correlation was analysed by Pearson’s correlation in (**b**,**c**). HFD, high-fat diet; ND, normal diet.

The diet change from HFD to ND led to a decrease in *Atf6α*, *Chop*, and *Erdj4* levels in the liver, implying attenuation of UPR during the 2 weeks of fat withdrawal. In contrast, the mean transcript levels of *Manf* (*p* = 0.003), *Grp78* (*p* = 0.039), and total *Xbp1 (tXBP1)* (*p* < 0.001) were increased in the HFD/ND group compared to the ND group (Fig. 4a). The levels of *sXbp1* (*p* = 0.006) remained high after the diet change in the HFD/ND group when compared with the ND group.

*Manf* transcript and protein levels correlated positively in the livers of the HFD/ND group (r = 0.79, *p* = 0.002, *n* = 12, Fig. 4b). Furthermore, *Manf* levels correlated positively with those of *Grp78* (r = 0.94, *p* < 0.001, *n* = 12, Fig. 4c) in the HFD/ND group. Similarly, a positive correlation between liver *Manf* and *Grp78* was found in the ND (r = 0.72, *p* = 0.009, *n* = 12, data not shown) and HFD (r = 0.83, *p* = 0.001, *n* = 12, data not shown) groups.

## Discussion

Dietary restriction regimes have been shown to optimize energy metabolism, prevent diseases, and promote healthy aging^6,7^. Recent research suggests that MANF is protective against age-related metabolic diseases as systemic MANF has been shown to relieve metabolic stress and age-related damage in mouse liver^30^. Another recent study suggested that MANF can regulate energy homeostasis, as transgenic overexpression of MANF in the brain hypothalamus led to overfeeding and obesity in mice^35^. In light of these new findings, and together with previous studies that have shown protective effects of MANF in animal models of neurodegenerative diseases^9,12–14^, we first aimed to determine if MANF is also induced upon health-promoting dietary restriction in humans. We examined the effects of therapeutic fasting on concentrations of circulating MANF and analysed correlations between MANF and clinical variables to reveal possible mechanisms related to the regulation of circulating MANF levels.

We observed that circulating MANF concentrations increased on average by 23% after therapeutic fasting in the human study group. Plasma MANF concentrations did not correlate with fasting glucose or fasting insulin, before or after dietary restriction, suggesting that circulating MANF levels are not directly linked to glucose homeostasis. In a previous study, circulating MANF levels were found to associate with insulin resistance in prediabetic type 2 diabetes subjects^17^. Similar to previous results from subjects with normal glucose tolerance, we observed no correlation between circulating MANF and insulin sensitivity indexes.

Interestingly, we found that plasma MANF levels and adiponectin measured before therapeutic fasting showed an inverse correlation in humans. Furthermore, the percentage change in MANF and adiponectin levels correlated inversely after fasting. In contrast to adiponectin, plasma MANF levels did not correlate with that of leptin or IGF-1, two other energy-metabolism regulating hormones^36,37^. Increased circulating concentrations of adiponectin are generally related to beneficial metabolic effects, such as improved insulin sensitivity and decreased levels of obesity and insulin resistance^4^. Although adiponectin levels did not change significantly after therapeutic fasting in the current human study group, an increased concentration of adiponectin has been reported after very low calorie diet^38^. The negative correlation between MANF and adiponectin levels observed in the human study population were also observed in mice. A trend towards a negative correlation between MANF and adiponectin was observed in mouse serum, and a clear negative association was detected between MANF protein concentrations in the liver and serum adiponectin. Adiponectin regulates glucose and lipid metabolism in the liver by suppressing glucose production and stimulating fatty acid oxidation^39^. Since systemic MANF likely regulates liver metabolism^30^, a possible connection between adiponectin and MANF signalling in the liver requires further studies.

To create a mouse model system, we subjected 13-week-old DIO mice to diet change from HFD to normal chow, thus withdrawing fat from the diet, and generated MANF knock-out tissue-verified ELISA for mouse MANF detection. After the diet switch, mouse weights reverted to normal within one week. In similar mouse studies, the weight reversal has been slightly slower. For example, diet change from HFD to low fat diet or normal chow of 16–17-week-old mice led to weight reversal within two weeks time^40,41^. Our mice were slightly younger at the time of diet switch, which may have affected the speed of weight loss. Variations in diet composition between different studies may also contribute to the time scale of weight reversal.

Following the change from a HFD to a ND, the average MANF concentration increased by 58% in serum and by 72% in the liver compared the HFD group. We found a positive correlation between liver and serum MANF concentrations in the HFD/ND group, suggesting that a proportion of circulating MANF originates from liver (the major source of plasma proteins) after diet change. In another study, a luciferase reporter construct, designed based on the functional properties of the ER retrieval sequence of MANF, was used for monitoring calcium depletion related to ER stress. The reporter showed increased secretion from the liver to plasma of rats during *ad libitum* HFD for 3 weeks, whereas caloric restricted HFD normalized the reporter levels indicating restoration of ER calcium balance in the liver^42^. In our study, serum MANF levels were unchanged in mice after HFD of 9 weeks. It is possible that the observed increase in reporter levels^42^ are due to short-term HFD. If so, circulating MANF might act during early adaption to metabolic changes. Serum MANF levels in our mice could have increased briefly during the development of weight gain and reverted to “normal” levels during the experiment. In the study by Sousa-Victor *et al*. plasma MANF levels were decreased in mice after 12 weeks of *ad libitum* HFD^30^. In future experiments, it would be informative to study the time course of MANF levels during HFD and diet intervention in order to better understand the regulation of circulating MANF. Furthermore, to investigate whether the observed increase in mouse MANF levels after diet change is caused by fat withdrawal or rather by energy restriction, it would be informative to measure MANF levels in mice subjected to caloric restricted HFD or ND.

Obesity and nutrient excess are linked to activated UPR in the liver of both humans and mice^43,44^. Since the expression of MANF is increased in ER-stress related disease models *in vivo*^22–25^, we investigated whether MANF expression in the liver was associated with ER stress and analysed the expression of UPR genes in the ND, HFD, and HFD/ND groups. Accordingly, our data show that UPR pathways via IRE1α, ATF6, and PERK sensors are activated in the livers of DIO mice on a HFD. Despite the activated UPR, liver *Manf* mRNA and protein levels were not significantly increased in the HFD group compared with the ND group. In future studies, additional experimental time points could reveal whether MANF levels are increased briefly during the adaptation to HFD.

After diet change from HFD to ND, mice weight decreased back to normal and levels of *Atf6a*, *Chop*, and *Erdj4* were significantly downregulated, indicating decreased ER stress in the liver. However, *sXbp1* levels remained high, suggesting active IRE1α signalling after dietary switch. It has been shown that hepatic IRE1α can function as a metabolic regulator activated by food deprivation^45^. After diet change, *Manf* mRNA levels were significantly upregulated in the HFD/ND group compared to the ND group. Since the transcription factors sXBP1 and ATF6α are known to induce MANF expression^19,20,46^, it is possible that MANF, downstream of IRE1α, is necessary for metabolic adaptations related to the diet change in the liver.

MANF is known to interact with glucose regulated protein 78 (GRP78, also known as BiP), which is a major ER chaperone that regulates the activation of UPR sensors^10,28^. A recent study suggested that MANF mechanistically stabilises GRP78 interaction with client proteins^47^. Accordingly, we observed a strong positive correlation between *Manf* and *Grp78* expression suggesting that changes in *Manf* expression can be related to the regulation of protein homeostasis and UPR. Ablation of MANF expression renders cells vulnerable to ER stress-induced cell death^18,24,48^, whereas overexpression of MANF^18^ or extracellularly applied MANF^26,49,50^ can attenuate ER stress and protect cells against ER stress-induced apoptosis *in vitro*. It remains to be studied whether the observed increase in MANF expression mediates the attenuation of ER stress and UPR signalling along with diet change in the mouse liver.

In summary, increased levels of MANF were observed after therapeutic fasting in humans and after diet intervention in obese mice, suggesting that endogenous MANF may be involved in promoting organismal health. Further studies will elucidate whether this knowledge can be used to design novel therapies. More specifically, it would be interesting to determine if the observed changes in MANF concentrations are directly related to changes in UPR activity, or if MANF is involved in metabolic regulation, possibly in connection to adiponectin.

## Methods

### Study population and intervention

Characteristics of the study population are presented in Table 1. The exclusion criteria included age <18 years, known type 1 or type 2 diabetes or pathologic OGTT, anorexia nervosa, underweight (BMI < 18.5 kg/m^2^), psychological disorders, malignancy, autoimmune diseases, and pregnancy. Overall, 40% (n = 16/40) of the population was overweight (BMI 25–29.9 kg/m^2^), and 35% (n = 14/40) was obese (BMI ≥ 30 kg/m^2^).

The intervention of therapeutic fasting was started with one preparation day, followed by on average 15.3 ± 6.3 days (range 9–49 days) of fasting. The number of fasting days was adjusted to individual goals and tolerance. During fasting, the participants received 300 to 400 kcal/day in the form of vegetable soup, fruit juice, and 20 g of honey. The fasting period was followed by at least 3 re-feeding days of progressive caloric intake. The criteria for discontinuation of fasting were any adverse advents, systolic blood pressure <90 mm Hg or diastolic <60 mm Hg, heart rate at rest >90 bpm, or subjective weakness.

Blood samples were collected before and after the fasting period. Pre-fasting samples were collected on the day before the first fasting day or on the first fasting day, and post-fasting samples on the last day of fasting. The blood samples were analysed for glucose, insulin, lipids, C-reactive protein (CRP), leukocytes, leptin, adiponectin, and IGF-1 levels. Additional samples were collected 0.5 h and 2 h after OGTT and analysed for glucose and insulin levels. For estimation of insulin resistance (IR) and beta cell function (%β), HOMA2 IR and HOMA2%β indexes were calculated with a non-linear HOMA-algorithm (Software Version 2.2.2. HOMA Calculator, University of Oxford). Overall changes in the parameters during fasting will be reported elsewhere (Drinda S., *et al*., manuscript under preparation).

The study was approved by the ethics commission of the medical council Baden-Württemberg (Germany) and is listed in the German register of clinical trials (DRKS-ID: DRKS00004249, date of registration 31/07/2012). The methods used were in accordance with relevant guidelines and regulations. All participants provided written informed consent.

### Mice and treatment

All animal experiments using mice were approved by the Finnish Animal Ethics Committee of the State Provincial Office of Southern Finland (licenses ESAVI/6005/04.10.03/2012 and ESAVI/10564/04.10.07/2014). All methods were carried out in accordance with the relevant guidelines and regulations. The mice were housed in a 12-h dark/light cycle and had *ad libitum* access to food and water. To study the effect of dietary intervention on MANF levels, mice were subjected to diet change from a HFD to a ND, thus reducing fat intake. Seven-week old DIO male C57BL/6J mice were acquired from Jackson Laboratories where the mice had been placed on a HFD at 6 weeks of age. The mice continued on a HFD (60% fat, 824054 RM AFE (M), Special Diet Services) until the age of 13 weeks. The mice then either remained on a HFD (*n* = 12) or were switched to a ND (4% fat, Teklad Global) (*n* = 12). A control group of mice (*n* = 12) received a ND only. Blood samples were collected from the saphenous vein at the age of 13 and 15 weeks. The mice were sacrificed at the age of 15 weeks and terminal blood was collected by cardiac puncture. Blood samples were left to coagulate for 30 min at room temperature and centrifuged at 3400 rpm for 10 min in tubes containing separation gel for serum collection. Liver, gastrocnemius muscle, and pancreas were dissected and snap frozen in liquid nitrogen. The sera and tissues were stored at −80 °C until analysis.

MANF knockout mouse sera and tissues used for the validation of mMANF ELISA were derived from conventional MANF KO mice, which were generated by breeding of *Manf*^+/−^ heterozygous mice. MANF (Manf_D06, C57BL/6N-*Manf*^* tm1a(KOMP)Wtsi*^) targeted embryonic stem cell clones were originally obtained from the KOMP Repository (http://www.komp.org)^15^.

### Tissue lysates

Tissues were ground by a Bessman Tissue Pulverizer (Spectrum™) precooled in liquid nitrogen. A part of the tissue crush was homogenised by plastic pestles in ice-cold lysis buffer [137 mM NaCl, 20 mM Tris-HCl, pH 8.2, 2.5 mM EDTA, 1% NP40, 10% glycerol, 0.5 mM Na_3_VO_4_, and Complete Mini protease inhibitor cocktail (Roche)] added as 100 μl per 10 mg of the tissue. The lysates were incubated on ice for 20 min, after which they were centrifuged at 12 000 rpm for 20 min at 4 °C. The supernatants were then collected and stored at −80 °C until analysis.

### Quantitative real-time PCR

Total tissue RNA was extracted from a maximum of 30 mg liver crush by a NucleoSpin® RNA (Macherey-Nagel) kit according to the manufacturer’s instructions. One microgram of DNase-treated RNA was reverse transcribed into cDNA by Maxima H minus RT (Thermo Fischer). 2.5 μl of cDNA was used in a qPCR reaction with a LightCycler® 480 SYBR Green I Master run in a LightCycler® 480 Real-Time PCR System (Roche). The primer sequences employed in this study are listed in Supplementary Table S6. Expression levels were calculated according to the primer efficiency values obtained from standard curves run on the same plate with samples^51^. Gene expression levels were normalised to the levels of β-actin analysed on the same plate.

### Human MANF ELISA

The development and validation of in-lab ELISA for the measurement of MANF in human blood has been described earlier^16^. Briefly, the human MANF sandwich ELISA is built on goat anti-human MANF coating antibody (AF3748, R&D Systems), and horseradish peroxidase (HRP)-conjugated mouse anti-human MANF detection antibody (4E12, Icosagen). The dynamic range of human MANF ELISA is 62.5–2000 pg/ml, and its sensitivity is 45 pg/ml. For the quantitation of human MANF in plasma, samples were diluted 1:20 and incubated with 0.5 mg/ml Immunoglobulin Inhibiting Reagent (Seralab) for 1 h on ice before adding them to the pre-coated ELISA plate.

### Mouse MANF ELISA

As the human MANF ELISA^16^ does not cross-react with mouse MANF, an ELISA was developed for the quantitation of mouse MANF in serum and tissues. Briefly, a 96-well plate (MaxiSorp, Nunc) was coated with goat anti-human MANF (AF3748, R&D Systems) antibody at a concentration of 2 μg/ml in 50 mM carbonate coating buffer (pH 9.6). After overnight incubation at 4 °C, the coated wells were washed with buffer (PBS/0.05% Tween) and blocked with 1% casein in PBS/0.05% Tween. Mouse sera were diluted 1:40 in blocking buffer and applied to the ELISA plate in duplicate, along with standard curve samples of recombinant mouse MANF (CYT-827, ProSpec) ranging from 31.25 to 1 000 pg/ml. MANF in mouse tissue lysate samples were quantified against the standard curve prepared from recombinant human MANF ranging from 62.5 to 1 000 pg/ml (P-101–100, Icosagen). The samples and standards were incubated over night at 4 °C. The next day, the wells were washed and rabbit anti-MANF (LS-B2688, LSBio) diluted in blocking buffer at 0.5 μg/ml was added to the wells and incubated for 3 h at 37 °C followed by incubation with a secondary HRP-linked donkey anti-rabbit antibody (NA9340V, GE Healthcare, 1:2000) for 2 h at room temperature. Bound HRP-linked antibody was measured by adding substrate (3,3′,5,5′-tetramethylbenzidine, DuoSet ELISA Development System, R&D Systems) and measuring the absorbance at 450 nm and 540 nm for wavelength correction.

The assay specificity, sensitivity, dynamic range, accuracy (% Relative Error, % RE = derived concentration/expected concentration × 100%) and precision [% CV, % CV = standard deviation (SD)/mean x 100%] were determined as described^52^. The sensitivity of the mouse MANF ELISA was determined by the mean response of 10 blank samples added with three standard deviations and calculating the value from the standard curve. Intra-assay variation was determined by measuring three samples of varying MANF concentration in replicates of 10 from different parts of a plate. Interassay precision was determined by running two different samples in duplicate on six independent assays. The specificity of the ELISA was tested by measuring cross-reactivity to mouse serum and tissue lysates from *Manf* knockout mice^15^ and to human recombinant CDNF (Icosagen). Linearity of dilution and recovery of spiked MANF in the study samples were analysed.

### Detection of haemolysis

Haemolysis in mouse sera was found to affect the results analysed by mMANF ELISA. Haemolysis in sera was analysed by absorbance at 414 nm^53^. All samples with Abs_414nm_ ≥ 0.3 were excluded from the analysis. MANF concentrations in human plasma samples as measured by human MANF ELISA showed no correlation with absorbance readings at 414 nm (data not shown). Overall, human samples gave very low absorbance values at 414nm (on average 0.09 ± 0.04), suggesting low or no presence of haemolysis.

### Mouse adiponectin

Adiponectin in mouse serum was quantified by Mouse Adiponectin/Acrp30 Quantikine ELISA Kit (MRP300, R&D Systems) according to the manufacturer’s instructions.

### Statistical analysis

Data are presented as mean ± SD. Shapiro-Wilk test was used to determine normality of distribution. Circulating MANF in humans showed a non-normal distribution, thus non-parametric tests were applied. Wilcoxon signed-ranks test was used to compare MANF levels in the paired samples taken before and after fasting. Correlation of MANF with different parameters was evaluated by Spearman’s rank correlation. In mouse samples, MANF and other parameters showed a normal distribution and parametric tests were thus applied. Differences in MANF levels between study groups were studied by one-way ANOVA followed by Tukey HSD post-hoc test and correlations by Pearson’s r. *p*-value < 0.05 was considered statistically significant. Statistical analysis was performed with SPSS 21.0.

## Supplementary information

Dataset 1

## Data Availability

The datasets generated during and/or analysed during the current study are available from the corresponding author on reasonable request.
