# Supplementary material for: Mesencephalic Astrocyte-Derived Neurotrophic Factor Is Upregulated with Therapeutic Fasting in Humans and Diet Fat Withdrawal in Obese Mice
Source: Sci Rep. 2019 Oct 4;9:14318. doi: 10.1038/s41598-019-50841-6 (PMC6778185; doi:10.1038/s41598-019-50841-6)
Supplement: Supplementary file 1 — Dataset 1 [file 41598_2019_50841_MOESM1_ESM.pdf]

Mesencephalic Astrocyte-Derived Neurotrophic Factor Is Upregulated with Therapeutic Fasting in Humans and Diet Fat Withdrawal in Obese Mice

Emilia Galli[1], Jari Rossi[2], Thomas Neumann[3,4], Jaan-Olle Andressoo[1,5,6], Stefan Drinda[7,8], Päivi Lindholm[1*]

[1]Institute of Biotechnology, Helsinki Institute of Life Science, University of Helsinki, Helsinki, Finland

[2]Department of Anatomy, Faculty of Medicine, University of Helsinki, Helsinki, Finland

[3]Department of Internal Medicine III, Friedrich Schiller University Jena, Jena, Germany

[4]Department of Rheumatology, Cantonal Hospital St. Gallen, St. Gallen, Switzerland

[5]Department of Pharmacology, Faculty of Medicine, Helsinki Institute of Life Science, University of Helsinki, Helsinki, Finland

[6]Division of Neurogeriatrics, Department of Neurobiology, Care Sciences and Society, Karolinska Institutet, Stockholm, Sweden

[7]Hospital Buchinger-Wilhelmi, Überlingen, Germany

[8]Department for Rheumatology, Clinic St. Katharinental, Diessenhofen, Switzerland

*Correspondence and requests for materials should be addressed to Päivi Lindholm (paivi.pulkkila@helsinki.fi)

Supplementary Tables

Sample	OD	pg/ml
WT mouse serum	0.369	340.6
KO mouse serum	0.005	8.2
WT mouse muscle	0.99	800.6
KO mouse muscle	0.005	8.2
WT mouse kidney	0.903	739.3
KO mouse kidney	0.001	2.0
WT mouse lung	1.025	825.0
KO mouse lung	0.004	6.8
Recombinant human CDNF (500 ng/ml)	0.003	5.3
**Standard (recombinant mouse MANF, pg/ml)**	**OD**	**pg/ml**
1 000	1.283	1001.8
500	0.594	514.5
250	0.256	248.1
125	0.111	120.4
62.5	0.050	60.6
31.25	0.025	32.7
0	0	
**Standard (recombinant human MANF, pg/ml)**	**OD**	**pg/ml**
1 000	1.278	993.1
500	0.464	513.4
250	0.150	245.7
125	0.051	122.1
62.5	0.019	63.6
0	0	

Supplementary Table S1. Specificity of mouse MANF ELISA. Concentration values measured for serum and tissue samples from MANF KO mouse[1], and for recombinant human CDNF tested at concentration of 500 ng/ml, were below the sensitivity level (29 pg/ml) of mouse MANF ELISA. In contrast, the ELISA gave high values to parallel samples from WT mouse, prepared and diluted identically to the MANF KO samples. WT, wild type; KO, knock out of MANF; OD, optical density.

Sample	Dilution	Average abs	mMANF std pg/ml	*DF %RE	hMANF std pg/ml	*DF %RE
Serum 1	1:5	0.423	430.4	100	546.3	100
	1:10	0.205	230.9	107	346.2	127
	1:20	0.081	104.1	97	193.2	141
	1:40	0.029	42.9	80	100.9	148
Serum 2	1:5	0.323	341.0	100	460.7	100
	1:10	0.176	202.5	119	314.5	137
	1:20	0.071	93.1	109	178.0	155
	1:40	0.025	38.1	89	92.4	160
Heart	1:250	0.453	407.1	100	505.8	100
	1:500	0.158	163.6	80	254.9	101
	1:1 000	0.054	64.2	63	126.2	100
	1:2 000	0.019	26.0	51	63.9	101
Pancreas	1:10 000	0.518	457.3	100	552.0	100
	1:20 000	0.175	178.4	78	272.1	99
	1:40 000	0.057	67.3	59	130.7	95
	1:80 000	0.018	25.3	44	62.7	91
Brain	1:500	0.505	446.8	100	542.5	100
	1:1 000	0.172	175.5	79	268.7	99
	1:2 000	0.054	65.0	58	127.3	94
	1:4 000	0.015	20.7	37	53.9	80

Supplementary Table S2. Dilutional linearity of endogenous MANF protein in serum and tissue lysates from wild type mouse. %RE were within 80-120% (highlighted in red) only if serum samples were analyzed using standard curve equation from recombinant mouse MANF and tissue samples using standard curve equation from recombinant human MANF. DF, dilution factor; % RE, % recovery (compared to the first dilution); std, standard.

Concentration of		Intra-assay variation			Interassay variation	
**recombinant mouse MANF**	N	Mean (pg/ml)	% CV	N	Mean (pg/ml)	% CV
Low	10	46.6	7.5	8	63.4	16.5
Medium	10	125.1	3.7	8	190.6	7.2
High	10	724.5	4.1	8	526.5	5.4
		**Average**	5.1		**Average**	9.7
**recombinant human MANF**	N	Mean (pg/ml)	% CV	N	Mean (pg/ml)	% CV
Low	10	114.5	8.8	6	60.3	13.4
Medium	10	313.4	13.0	6	240.2	4.7
High	10	823.4	7.5	6	730.0	7.7
		**Average**	9.8		**Average**	8.6

Supplementary Table S3. Intra-assay precision (repeatability) and inter-assay precision (reproducibility) of the mouse MANF ELISA. Results are shown as a mean of ten replicates for the intra-assay precision, and as a mean of six to eight separate assays for the inter-assay precision. % CV, % coefficient of variation.

Dilution	1st	2nd	3rd	2nd	3rd
**mMANF in std**	**Measured concentration (pg/ml)**			***DF, %RE**	
WT serum 1	270.5	132.6	63.3	98	94
WT serum 2	392.6	185.1	78.3	94	80
WT serum 3	262.5	119.1	52.9	91	81
WT serum 4	279.8	129.4	55.7	92	80
WT serum 5	337.5	166.7	70.8	99	84
KO serum 1 + rec. MANF	475.2	216.1	103.2	91	87
KO serum 2 + rec. MANF	420.7	219.2	112.1	104	106
	**Mean recovery (%)**			**96**	**87**
**hMANF in std**	**Measured concentration (pg/ml)**			***DF, %RE**	
WT Liver	146.0	79.1	44.7	108	under LLOQ
WT Pancreas	704.4	382.2	196.9	109	112
WT Skeletal muscle	731.2	402.7	195.5	110	107
WT Hypothalamus	426.0	191.4	102.7	90	96
WT Cortex	716.2	377.3	180.3	105	101
	**Mean recovery (%)**			**104**	**104**

Supplementary Table S4. Linearity of dilution (%) of MANF in serum and tissue samples. Dilutional linearity of spiked recombinant MANF was assessed in serum from MANF KO mouse. Dilutonal linearity of endogenous MANF was assessed in sera and tissue lysates from wild type mouse. Samples were diluted sequentially by 1:2. The recovery in 2nd and 3rd dilution is calculated compared to the measured concentration in the 1st dilution. All results were within 80-120% RE. DF, dilution factor; % RE, % recovery (compared to the first dilution); mMANF, mouse MANF; hMANF, human MANF; std, standard; WT, wild type; KO, knock out of MANF; rec., recombinant; LLOQ, lower limit of quantification (62.5 pg/ml, when using hMANF in the standard curve).

	Measured concentration (pg/ml)				Endogenous MANF subtracted, %RE		
**Spike (mMANF, pg/ml)**	**-**	**50**	**100**	**250**	**50**	**100**	**250**
WT serum 1	130.5	184.5	231.6	385.2	108	101	102
WT serum 2	163.9	219.4	261.0	412.5	111	97	99
WT serum 3	132.7	177.2	220.0	376.6	89	87	98
KO serum 1	n.d.	47.8	90.5	221.9	96	90	89
KO serum 2	n.d.	43.4	85.2	218.7	87	85	87
KO serum 3	n.d.	48.9	103.6	236.5	98	104	95
				**Mean (±SD) recovery %**		**96±8**	
**Spike (hMANF, pg/ml)**	**-**	**100**	**150**	**250**	**100**	**150**	**250**
WT liver	126.7	-	278.1	-	-	101	-
WT skeletal muscle	580.3	-	-	810.2	-	-	92
KO brain	n.d.	101.6	-	265.2	102	-	106
KO skeletal muscle	n.d.	95.2	-	239.9	95	-	96
				**Mean (±SD) recovery %**		**99±5**	

Supplementary Table S5. Recovery (%) of spiked recombinant mouse MANF (mMANF) in wild type and MANF KO mouse sera diluted 1:40, and the recovery of recombinant human MANF (hMANF) in diluted tissue lysates. All results were within 80-120% RE. WT, wild type; KO, knock-out of MANF.

Target	Forward	Reverse
Manf	gacagccagatctgtgaactaaaa	tttcacccggagcttcttc
Dnajb9/Erdj4	taaaagccctgatgctgaagc	tccgactattggcatccga
Grp78/BiP	ataaaccccgatgaggctgt	catcaagcagtaccagatcacc
tXbp1	caccttcttgcctgctggac	gggagccctcatatccacagt
sXbp1	gagtccgcagcaggtg	gtgtcagagtccatggga
Atf6α	ggacgaggtggtgtcagag	gacagctcttcgctttggac
Ddit3/Chop	ccaacagaggtcacacgcac	tgactggaatctggagagcga
Atf4	atggccggctatggatgat	cgaagtcaaactctttcagatccatt

Supplementary Table S6. Used primer sequences for mouse liver qPCR. Primer sequences for Grp78, total (t)Xbp1, spliced (s)Xbp1, Atf6a, Ddit3/Chop, and Atf4 were derived from Lindahl *et al*. (2014)[1]. Dnajb9/Erdj4 primer sequences were from Cubillos-Ruiz *et al*. (2015)[2].

a

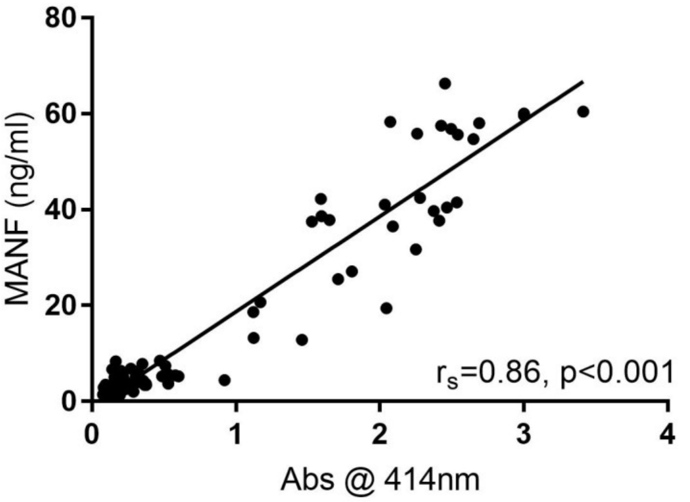

b

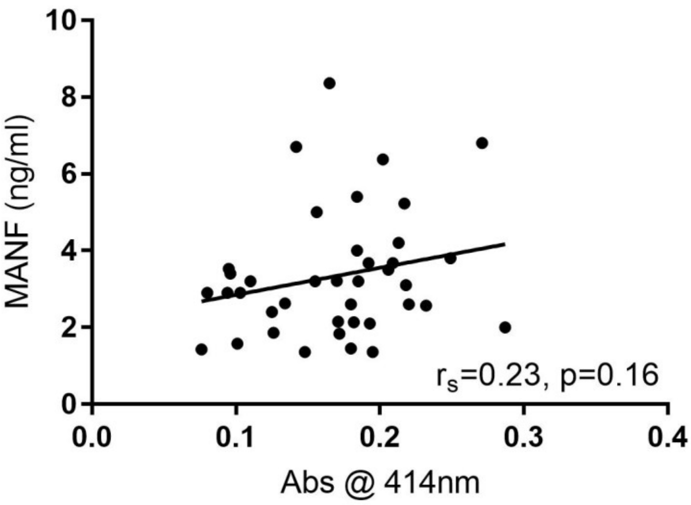

Supplementary Figure S1. Higher mouse MANF ELISA readings in haemolysed mouse blood. a) Mouse MANF serum concentration measured by mMANF ELISA strongly correlated with the absorbance values at 414 nm, signifying the extent of haemolysis (r_s=0.86, *p*<0.001, *n*=90). b) When samples with absorbance values at 414 nm equal or above 0.3 were excluded, the positive correlation was abolished (r_s=0.23, *p*=0.16, *n*=39). Only blood samples showing Abs_{414nm} < 0.3 were included to the study.

Supplementary references

1 Lindahl, M. *et al.* MANF is indispensable for the proliferation and survival of pancreatic beta cells. *Cell reports* **7**, 366-375, doi:10.1016/j.celrep.2014.03.023 (2014).

2 Cubillos-Ruiz, J. R. *et al.* ER Stress Sensor XBP1 Controls Anti-tumor Immunity by Disrupting Dendritic Cell Homeostasis. *Cell* **161**, 1527-1538, doi:10.1016/j.cell.2015.05.025 (2015).
